# The effectiveness and sustainability of supervised balance training in chronic ankle instability with grade III ligament injury: a one-year prospective study

**DOI:** 10.1186/s13047-022-00514-x

**Published:** 2022-02-01

**Authors:** Zong-chen Hou, Hong-shi Huang, Ying-fang Ao, Yue-lin Hu, Chen Jiao, Qin-wei Guo, Xin Miao, Nan Li, Yan-fang Jiang, Dong Jiang

**Affiliations:** 1 Department of sports medicine of Peking university third hospital, Institute of Sports Medicine of Peking University, Beijing Key Laboratory of Sports Injuries, No.49 North Garden Road, Haidian, Beijing, 100191 China; 2 Research Center of Clinical Epidemiology, Peking University Third Hospital, No.49 North Garden Road, Haidian, Beijing, 100191 China

**Keywords:** Chronic ankle instability, Rehabilitation, Plantar pressure, Muscle strength

## Abstract

**Background:**

To determine the effectiveness and sustainability of supervised balance training in people with chronic ankle instability (CAI) with grade III ligament injury.

**Methods:**

Twenty young adults (12 males and 8 females) diagnosed with CAI with grade III ligament injury underwent 3 months of supervised balance training. The self-reported functional questionnaire, plantar pressure (walking and single leg standing), and isokinetic ankle strength were consecutively evaluated at pre-training, 3 months, 6 months and one year. Paired T tests were used to explore changes in muscle strength and plantar pressures following the supervised balance training. According to whether the patient had sprain recurrence, the patients were divided into sprain recurrence group and control group. The risk factors of sprain recurrence were explored with univariate analysis and multivariable logistic regression.

**Results:**

The self-reported functional scores, the plantar pressure distribution and the muscle strength showed significant immediate improvements after 3 months of supervised balance training. At 6 months post-training, peak force under 2nd metatarsal, time to peak force under the medial hindfoot, time to boundary measurements and dorsiflexion, and eversion strength were partly declined to the pre-training level. 16 patients (80%) resumed the daily life and sports without sprain recurrence during the follow-up. Four patients (20%) reported ankle sprain during the follow-up, and the sprain recurrence group showed significantly higher Beighton scores (*p* = 0.012) and weaker initial inversion strength (*p* = 0.022) than the control group.

**Conclusions:**

Three months’ of supervised balance training could effectively improve postural control and muscle strength of CAI cases with grade III ligament injury, although these improvements would partially deceased over time. Additional strength exercises for dorsiflexion and eversion should be supplemented from 6 months. Higher Beighton score and initial inversion muscle strength weakness might increase the risk of sprain recurrence.

**Trial registration:**

ChiCTR, ChiCTR1900023999, Registered 21 June 2019, https://www.chictr.org.cn/edit.aspx?pid=39984&htm=4

**Supplementary Information:**

The online version contains supplementary material available at 10.1186/s13047-022-00514-x.

## Introduction

Lateral ankle sprain is a common musculoskeletal injury in sports [1]. Although most patients resume daily life after the primary sprain, about 34% of individuals can suffer from chronic ankle instability (CAI), which is characterized as recurrent sprain, episodes of giving-way of the ankle joint, pain, deficits of postural control and muscle strength [1]. Treatment strategy includes conservative or operative solutions, mainly depending on the severity of ankle sprains, which are classified from grades I to III (mild, moderate, or severe) [2]. Conservative treatment is more effective for CAI patients with Grade I and Grade II ligament injuries while treatment for chronic ankle instability (CAI) with grade III injuries is controversial [3]. Surgery is commonly recommended to CAI patients, especially to those with combined intra-articular lesions (osteochondral lesions (OCLs), osteophyte, impingement, loose body, etc.) causing obstructive symptoms [4–6]. However, considering the invasiveness and potential complications of the surgery, conservative treatment could be the first choice for CAI cases with isolated lateral ankle ligament but without intra-articular lesions.

Balance-training programs have been shown to be effective in improving postural control and muscle strength in CAI patients [7] in the short term. However, some research suggests that the effectiveness of conservative treatment (such as subjective symptoms [8] and eversion muscle strength [9]) might be temporary, and about 21.4% of CAI patients still had re-sprains [8] and the postural stability deficits at 6 months post-intervention [9]. However, previous studies merely targeted some single treatment (such as resistance tubing [8] and wobble board [9]), and the follow-up period was only 6 months. In addition, those studies included CAI cases with varying degree of ligament injury but did not distinguish the CAI cases with grade III ligament injury. At present, there is limited evidence for long-term effectiveness and continuity of systematic rehabilitation training for CAI patients with grade III ligament injury.

In terms of the post-training functional evaluation, most of the studies focused on the subjective feeling instead of objective evaluation, thus, make it difficult to guide clinical practice. In fact, the foot pressure measures, such as center-of pressure (COP) excursion, time-to-boundary (TTB) and peak plantar analysis have been commonly used to identify the postural control deficits of those with CAI. CAI cases have significantly less time to make postural corrections to meet the stability demands [10] and a significantly increased lateral loading [11] compared with healthy controls. These objective parameters might be important predictors for the effectiveness and sustainability and help treatment decision making.

In the present study, 20 CAI patients with isolated lateral ankle ligament injury were included and accepted 3 months of supervised balance training and consecutive follow-up for 1 year. The purpose of the present study was to determine the mid-term effectiveness and the sustainability of a balance training program and preliminarily explore the risk factors of sprain recurrence in the CAI cases. We hypothesized that balance training would improve the muscle strength, foot pressure distribution and postural stability, but some improvements would be weakened with time. These results may help us optimize rehabilitation strategies to improve the effectiveness of balance training for the CAI cases with grade III ankle ligament injury.

## Materials and methods

### Design

From September 2018 to April 2019, 20 CAI patients who were diagnosed as grade III ligament injury and ready for rehabilitation were included in the study. A priori power analysis was completed using data from a previous study in which the researchers examined the effects of a similar balance-training program [12]. The study was approved by the IRB Medical Committee of our hospital (IRB00006761-M2019164) and the written consent was obtained from all patients. The study was registered in Chinese Clinical Trial Registry (ChiCTR), and the number was ChiCTR1900023999.

### Patient enrollment

The inclusion criteria were (i) aged 18 to 40 years, (ii) unilateral injury with a history of at least one significant lateral ankle sprain (at least 12 months prior to study enrolment) that caused inflammatory symptoms and disrupted activity, (iii) the most recent ankle sprain occurred > 3 months prior to study participation, (iv) reports of the previously injured joint “giving way” and/or recurrent sprain and/or “feelings of instability” (v) scoring < 24 on the Cumberland Ankle Instability Tool (CAIT) [13], (vi) the first time sprain was diagnosed as grade III [14, 15] injury of anterior talofibular ligament (ATFL) and/or calcaneofibular ligament (CFL) confirmed by both MRI and positive anterior drawer test (increased translation of 3 mm compared to the uninjured side or an absolute value of 10 mm of displacement) [16] and talar tilt test (10° of absolute talar tilt or 5° difference compared to the contralateral side) [17] by TELOS SD 900 Stress Device (Austin & Associates, inc. USA). All patients presented without a history of neurological or orthopedic impairment. Patients with combined intra-articular lesions (OCLs, osteophyte, impingement, loose body, etc.), any history of surgery, fracture requiring realignment and/or acute injury to the musculoskeletal structures (bone, joint structure and/or nerve) in either lower limb were excluded.

Upon enrollment, all the patients’ basic information was collected and evaluated, including gender, age, height, weight, involved side, pre-duration, sprain time and the Beighton score (Table 1). The Beighton score ≥ 4 was defined as the generalized joint hypermobility (gJHM). The postintervention data-collection session occurred within 48 h after the intervention ended. A follow-up session was performed 3 months, 6 months and 1 year since the pre-intervention data-collection session. Participants were instructed to cease all interventions during the follow-up session. During each data-collection session, we administered the patient-oriented outcomes (Foot and Ankle Ability Measure (FAAM), Cumberland Ankle Instability Tool (CAIT)) before evaluating the disease-oriented outcomes (isometric ankle strength, foot pressure and static and dynamic postural control).

**Table 1:** Participants’ information

Characteristics (*n* = 20)	Values
Age, y	26.4 ± 5.2
Height, cm	167.1 ± 8.4
Weight, kg	60.3 ± 9.5
No. of sprains	8.2 ± 4.1
Months since last sprain	21.1 ± 21.6
Sex, men: women	12: 8
Beighton score	3.3 ± 2.3
CAIT	15.4 ± 5.3
FAAM ADL	67.6 ± 10.3
FAAM sport	55.9 ± 11.4

*FAAM* Foot and Ankle Ability Measure, *CAIT* Cumberland Ankle Instability Tool

### Plantar pressure and posture control evaluation

The patients underwent three trials of single-limb stance on each leg with eyes closed on a force plate (AMTI; Watertown, MA, USA) for 10s [10, 18]. COP data were calculated from the three-dimensional force and moment signals and sampled at a rate of 50 Hz^18^. Subjects were instructed to stand as still as possible during testing with arms folded across their chests, holding the opposite limb at approximately 45° of knee flexion and 30° of hip flexion in accordance. If a subject touched down onto the ground, contacted the stance limb, or was unable to maintain standing posture during the 10-s trial, the trial was terminated and repeated. TTB measures and COP measures were computed separately in the ML and the AP directions using previously described methods and smaller TTB and larger COP indicated shorter time to reach the balance boundaries and more risk to fall [10, 18].

Following the standard calibration trial, the subjects were asked to walk barefoot at a self-chosen, comfortable, moderate velocity along the walkway. All subjects were allowed to familiarize themselves with the procedures before data collection. Subjects walked six times over the pressure plate (Footscan, RSscan International, Olen, Belgium) with 120 Hz sampling rate [19]. Three valid left and three valid right stance phases were measured. A trial was considered to be valid when the following criteria were met: stance phase registered as a heel strike pattern and no visual adjustments in step length or step frequency to aim on the pressure plate. Each print consisted of a time peak-force curve for eight regions of interest on the foot. The regions of interest, which were analyzed automatically by the system software, were medial heel (HM), lateral heel (HL), 1st to 5th metatarsal heads (M1 ~ M5), and toes (T1). The time variables were calculated as the ratio of time from the start of the stance to peak force under the region of interest and the total stance time (%). The peak force variables were calculated as the ratio of the peak force under the region of interest and were normalized by the body weight (N/kg) [20].

### Isokinetic strength measurement

As described in TW Kaminski’s research [21], isokinetic strength was assessed with a Biodex isokinetic dynamometer (Biodex Medical Systems Inc., Shirley, NY). Each subject’s foot was securely fastened on the Biodex chair, with the hip angle 80° flexion (0° neutral position) and 20°to 30° of knee flexion. Each subject was allowed three submaximal (50% capacity) warm-up repetitions at each velocity to become familiar with the isokinetic test procedure, then performed three maximal concentric test repetitions at 60 and 120°/s on both ankles. The resting interval was approximately one minute between tests for each motion, velocity, and side. At the end of testing, peak torque data were extracted from the torque curves.

### Balance training protocol

As shown in Table 2, the detailed balance training protocol was designed based on the widely used protocol from previously published papers [22, 23]. The protocol includes single-legged stance, wobble board, resistant band and hop exercises. All participants underwent the 3-month balance training intervention. The progressive balance-training program consisted of 24 supervised training sessions (two 60-min sessions per week).

**Table 2:** The balance training protocol

Exercise	Description	Progression
Single-legged stance	Performed up to 60s per repetition for up to 3 repetitions. Performed with eyes opened and eyes closed	Progressed when participants could complete a 60-s trial without a loss of balance. Increased no. of repetitions by 1 Changed surface from floor to using the Dyna-Disc^a^.
Wobble board	Slowly moved the board in the plantar- flexion/dorsiflexion and inversion/eversion directions without letting the board contact the floor. Performed up to 10 repetitions in each direction.	Progressed when participant could complete the task without upper extremity support. Added rotational directions.
Steamboats	Tied a 48-in Thera-band around the unstable ankle. Positioned stance foot 27-in from where Thera-band was tied. Performed up to 3 sets of 15 repetitions in each direction (hip flexion, extension, abduction, adduction).	Progressed when participants could complete the repetitions without a loss of balance or fatigue. Increased no. of repetitions from 10 to 15. Progressed to next level of resistance with the Thera-band.
Single-legged hop	Hopped as far as comfortable in the anterior direction. Performed up to 15 repetitions.	Progressed when participants could perform the task with minimal ankle and hip motion and no loss of balance on landing. Increased no. of repetitions from 5 to 10 to 15. Encouraged increased distance to participants’ tolerance. Progressed to medial, lateral, and posterior directions.
Quadrant hop	Hopped in numbered squares clockwise and counterclockwise while maintaining single-legged stance.	Progressed when participants could complete 2 sets of 5 hops without a loss of balance or fatigue Made unanticipated directional changes where investigator randomly called out numbers.
Single-legged ball catch	Performed up to 3 sets of 20 tosses	Progressed when participants could perform 20 tosses without a loss of balance. Tossed ball outside participants base of support. Performed during stance on a DynaDisc.
Toe touch down	Maintained single-legged stance on a step while lowering the unstable ankle in the anterior, posterior, medial, and lateral directions until the foot contacted floor. Performed up to 3 sets of 10 repetitions.	Progressed when participants could complete all trials without a loss of balance and with good lower extremity alignment (no eversion collapse) Increased no. of repetitions from 5 to 10. Increased height of step from 4 in to 12 in in 2-in increments
Hop ups and downs	Hop off step and landed in single-legged stance on floor. Performed up to 3 sets of 10 repetitions.	Progressed when participants could complete all hops without a loss of balance or fatigue. Increased no. of repetitions from 5 to 10. Increased height of step from 4 in to 12 in in 2-in increments Changed direction of hop. Hopped up onto step.

^a^ Exertools, Petaluma, CA. ^b^ The Hygenic Corporation, Akron, OH

### Risk factors analysis

Based on the ankle sprain recurrence after the balance training program through one year, the patients were divided into the sprain recurrence group and control group. The pre-training variables for each patient was used to analyze the risk factor for the sprain recurrence after the balance training.

### Data analysis

The self-reported function (FAAM and CAIT), postural control measures (TTB measures and COP-based measures, plantar pressure measures) were analyzed separately at pre-training, post-training, 3 months, 6 months and 1 year. Paired sample t test was used to assess changes in the dependent measures before and after balance training. Shapiro-Wilk test was used to assess normality of data. Group comparison was only conducted after the formation of both groups (1 year). Univariate analysis and multivariable logistic regression models were used to explore the risk factors of sprain recurrence after the balance training, alpha level was set a priori at *p* < 0.05. An a priori power analysis was completed using data from a previous study [23] in which the researchers examined the effects of a similar balance-training program. Based on an α level of .05, a power of 0.95, and an effect size of 0.97 determined by the FAAM-Sport, 16 participants were needed. Therefore, we enrolled 20 participants to account for up to 20% attrition.

## Results

### Self-reported questionnaire

**Fig. 1:**
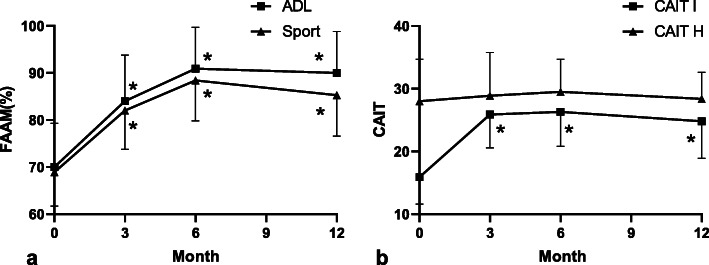
Pre-training and post-training scores of the FAAM and CAIT. (a), (b) represents changes in the FAAM and CAIT respectively. ^*^ indicated *p* < 0.05 for comparisons within the pre-training and each time point of post-training

The changes of self-reported function questionnaires are presented in the Fig. 1. Compared with the pre-training values there was a significant increase for the FAAM ADL (*p* < 0.05) and FAAM Sport (*p* < 0.05) and CAIT (*p* < 0.05) at all follow-up timepoints. Numbers of recurrent sprains decreased from 3.29 ± 2.87 to 1.63 ± 2.02 at one-year follow-up. However, there were four patients (20%) that still reported a sense of instability with at least once sprain recurrence while other 16 patients (80%) patients went back to pre-injury sport and general life.

### Plantar pressure distribution and postural stability

**Fig. 2:**
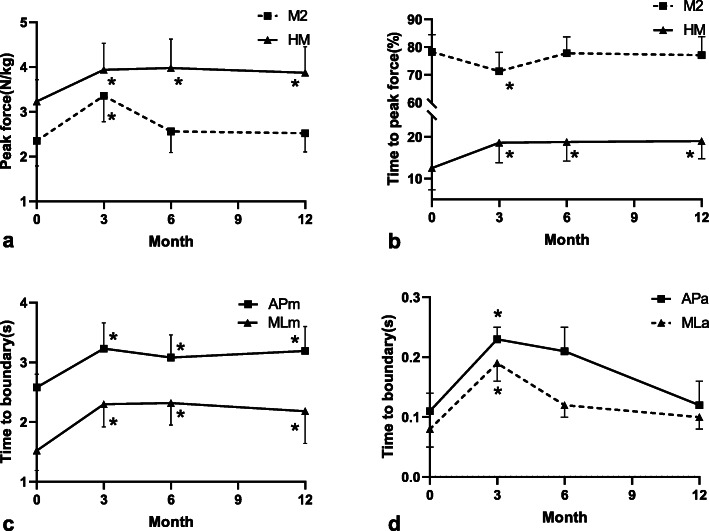
Measurements during the walking (a, b) and the single limb standing (c, d). TTBML (AP)m: mean value of the minimum time to boundary in the medial-lateral (anterior-posterior) direction, TTBML (AP)a: absolute minimum time to boundary in the medial-lateral (anterior-posterior) direction. HM, Medial heel; M2, the 2nd metatarsal heads. Dotted line indicates the declining variables after the balance training. ^*^ indicated *p* < 0.05 for comparisons within the pre-training and each time point of post-training

**Fig. 3:**
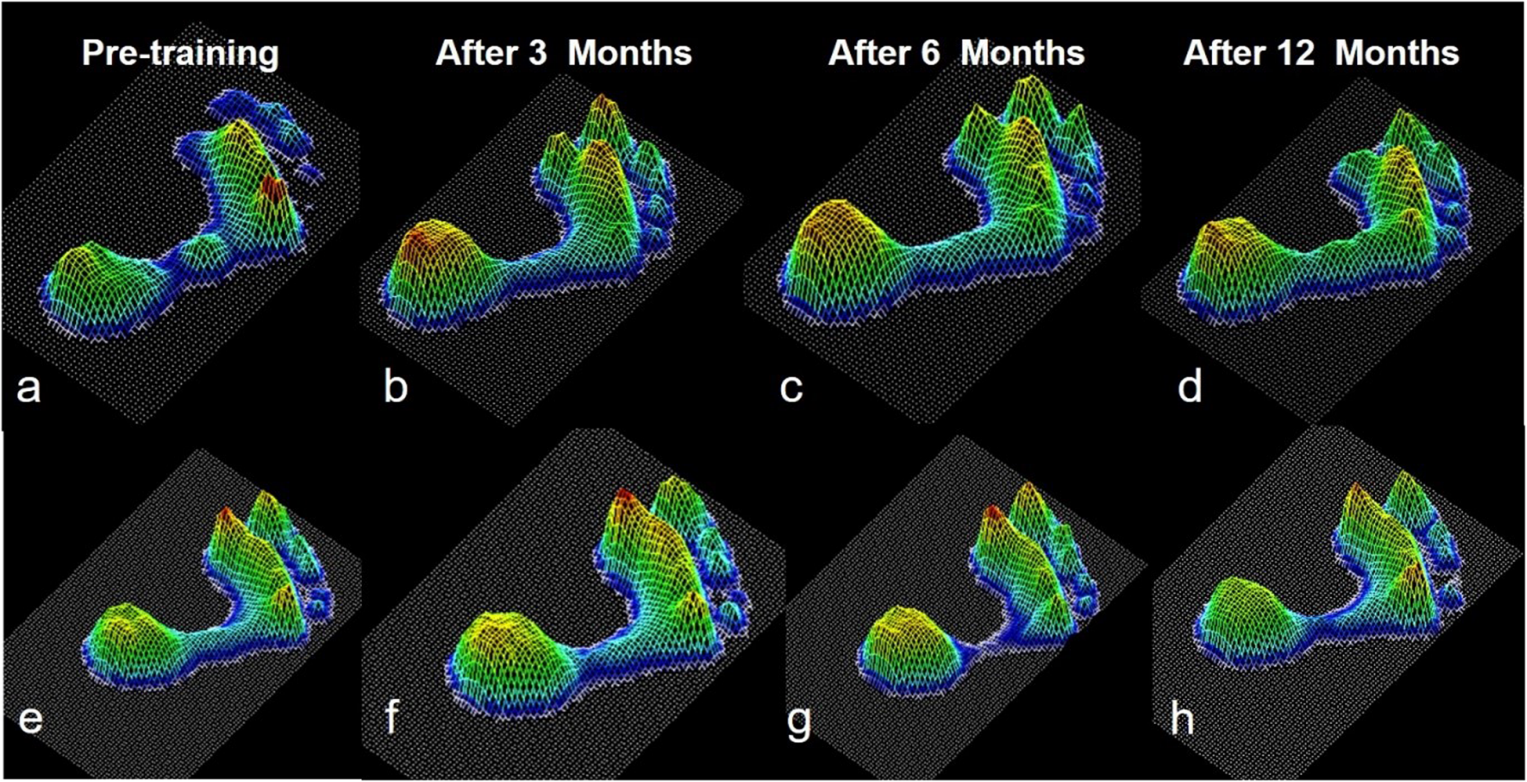
Foot pressure distribution changes of the patient from respective control group(a ~ d) and sprain recurrence group(e ~ h) in three-dimension model (Screenshot from footscan 7.0 software). The figures showed the peak force during the walking at pre-training, 3, 6 and 12 months post training. After the balance training, the foot distribution in the control group turned to the medial side of foot(a ~ d) while the foot distribution in the sprain recurrence group turned to the medial side then reversed to lateral side of foot again (e ~ h)

During walking, a significant increase in the peak force under the M2 (2.4 ± 1.2 vs 3.2 ± 1.9 N/kg, T = 3.79, *p* = 0.011) and HM (3.2 ± 0.9 vs 4.0 ± 1.2 N/kg, T = 3.99, *p* = 0.034) was observed at 3 months follow-up, and the changes of peak force in HM (3.6 ± 0.9 vs 5.0 ± 1.7, T = 4.03, *p* = 0.032) lasted until 6 months (Fig. 2. a, b). For the time to peak force (TPF), there was a significant decrease in M2 (79.5 ± 8.5 vs 72.3 ± 7.9%, T = 2.98, *p* = 0.031) and increase in HM (12.6 ± 4.6 vs 19.1 ± 4.9%, T = -2.65, *p* = 0.047) only at 3 months follow-up. No other differences were found at 6 months follow-up compared with the pre-training value. The foot pressure distribution changes in a three-dimension model of two individuals from respective control group and recurrence group are shown in the Fig. 3.

During single leg standing, TTB measurements were found to be significantly different with the pre-training level after 3-month training (Fig. 2. c, d). After 3 months’ balance training program, TTBAPa (T = 3.58, *p* = 0.02) and TTBMLa (T = 3.36, *p* = 0.02) temporarily increased and then decreased to the pre-training level while TTBAPm and TTBMLm showed continued improvement at 6 months and one-year (*p* < 0.05). There were no significant differences for the COP measures at all time points (*p* > 0.05).

### Muscle strength

**Fig. 4:**
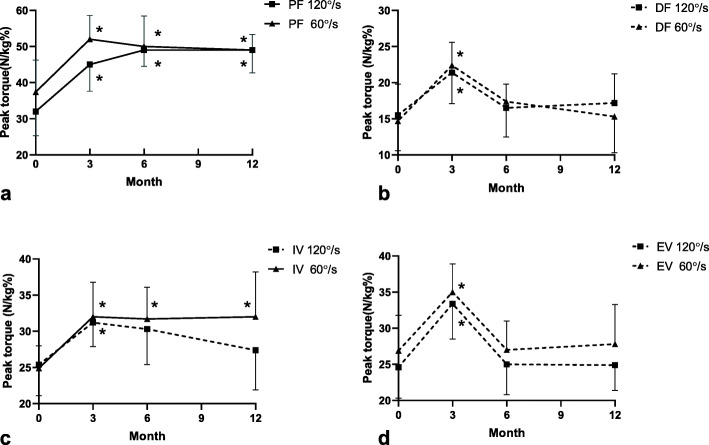
Measurements of isokinetic contraction muscle strength. Dotted line indicates the declining muscle strength after the balance training. PF, plantarflexion; DF, dorsiflexion; EV, eversion; IV, inversion. ^*^ indicated *p* < 0.05 for comparisons within the pre-training and each time point of post-training

As shown in Fig. 4, all the isokinetic contraction strength was significantly improved after 3 months of balance training (*p* < 0.05). Continuously significant increase in the 120°/s, 60°/s PF and 120°/s IV strength was observed at the one year follow up (*p* < 0.05), but the dorsiflexion and eversion muscle strength decreased to the pre-training level (*p* > 0.05) after six months until one year. Besides, 60°/s inversion strength decreased slightly after one year.

### Risk factors of sprain recurrence

To preliminarily explore the potential risk factors of recurrence, the univariate analysis and multivariable logistic regression model were used. Based on whether the scores < 24 CAIT or there was an obvious ankle sprain during the follow up, the patients were divided into the sprain recurrence group (*n* = 4) and the control group (*n* = 16). The baseline values in those two type individuals were analyzed with univariate analysis. Significant differences were found in the 60°/s inversion strength, Beighton scores, peak force under first metatarsal, medial hindfoot and lateral hindfoot (Appendix file 1, all *p* < 0.05). Using univariate analysis results (variables *p* < 0.1) and forward selection to create a multivariable logistic regression model, we found that the lower 60°/s isokinetic inversion muscle strength (OR 1.46; 95% CI, 1.23–1.98; *p* = 0.012) and a higher Beighton score (OR 1.27; 95% CI, 1.03–1.32; *p* = 0.032) could be the risk factors (Table 3).

**Table 3:** Final logistic regression model for the relationship between baseline variables and sprain recurrence at 1-year follow-up

	Sprain recurrence individuals (*N* = 4)	Control individuals(*N* = 16)	Odds radio	95% CI	*P* value
Beighton	4.33 ± 1.53	1.43 ± 1.62	1.27	1.03–1.32	0.032*
60°/s IV(N*m/kg)	13.47 ± 1.10	18.27 ± 2.09	1.46	1.23–1.98	0.012*
PF M1(N/kg)	2.77 ± 0.54	2.55 ± 1.05	1.02	0.88–1.13	0.322
PF HM(N/kg)	5.53 ± 1.18	5.47 ± 1.21	0.98	0.82–1.26	0.241
PF HL(N/kg)	4.44 ± 1.29	4.31 ± 1.34	0.85	0.67–1.23	0.085

CI, confidence interval. PF M1, HM and HL, Peak force under the 1st metatarsal heads, and medial hindfoot and lateral hindfoot. * indicates *p* < 0.05

## Discussion

The most important finding of the present study was that the 3 months of supervised balance training significantly improved self-reported function, postural control, and muscle strength for CAI patients with isolated ligament injury and most CAI patients with grade III ligament injury (80%) had satisfactory outcomes. However, some improvements on postural control and muscle strength declined after 6 months and 1 year. Additional strength exercises for dorsiflexion and eversion should be supplemented from 6 months. The generalized joint hypermobility (gJHM) and the initial inversion strength weakness could be the potential risk factors for sprain recurrence after balance training.

According to our results, most CAI patients with grade III ligament injury had satisfactory outcomes after the balance training. Similar improvements in self-reported function questionnaire [24, 25] and muscle strength [26, 27] were also found, but none of them investigated the long-term effectiveness of balance training and distinguish the severity of the ligament injury. The results of the present study implicated that the balance training is still effective even for patients with severe ligament injuries in the long term (i.e., up to 1 year). Although the ligaments are completely disrupted in the acute phase and the joints are obviously unstable in the chronic phase, the feeling of instability is partly improved by increasing the muscle strength and the posture control. However, we found that the strength improvement in dorsiflexion and eversion disappeared at the 6 months follow-up and a similar situation occurs with 60° eversion muscle strength at 1 year. It could be inferred that balance training will provide a short-term muscle strength improvement and additional strength exercises for dorsiflexion and eversion from 6 months and inversion exercise from 1 year might enhance and maintain the effect of balance training again. Further studies are needed to improve the balance training program for CAI cases.

Regarding the plantar pressure evaluation, we found a significant change in the TTB related measures during single leg standing. TTB related measurements involved a spatiotemporal analysis of COP data points, which was a novel approach to assessing postural control deficit in single-limb stance [10, 28]. It quantified the theoretical amount of time an individual had to use to make a postural correction to maintain postural stability. The results indicated that the grade III CAI patients needed longer time to reach the balance boundaries and had a lower risk to fall after the balance training. During walking, we found that the TPF of HM was more delayed and M2 came earlier after the balance training, which means a shorter duration of contact of the heel to central forefoot. The longer duration indicated a slowing down of weight transfer from heel strike to toe off, which was a sign of walking stability improvement [20]. The results also showed that greater loading under the M2 and HM which indicates a medial shift of the COP so as to decrease the susceptibility of ankle sprain [11]. However, improvements on stability during single leg standing and pressure distribution during walking was declined gradually over time.

The present study showed that the sprain recurrence patients had significantly higher Beighton scores, which indicated that the patients with gJHM might have poor rehabilitation effect for balance training. Previous studies [29, 30] have reported generalized joint hypermobility was an intrinsic risk factors related to recurrent lateral ankle sprain, so the hypermobility of ankle joint structure might increase the risk of ankle injury during the rehabilitation process. Similar poor outcomes have been reported for surgery treatment, such as the modified Broström procedure [16, 31]. In those cases, the repaired ligaments would eventually stretch and the patients need a longer period for lateral ligament recovery. However, the impact of gJHM on conservative treatment effect was rarely reported. In theory, the increased muscle strength brought by functional training can enhance joint stability. However, the joint capsule of these patients with gJHM may be compromised, so that the improvement of muscle strength is not enough to restore sufficient stability. This may be the reason for the relatively poor results of these patients. Considering the limited number of cases, the effect and the mechanism of joint hyper-relaxation on conservative training requires further controlled studies.

In this study, we also found the relatively weaker 60°/s inversion strength could be another risk factor of the failure. Several studies have shown that subjects with CAI exhibited strength deficits in their invertor musculature [32–34]. It is suggested that the ankle joint invertors play an integral role in controlling the rate of calcaneal eversion as the body’s center of mass is displaced laterally beyond the base of support [35]. The inversion strength weakness lead in uncontrolled weight transfer to the lateral side of the foot during single leg standing, thus increased frequency of ankle inversion episodes. For patients with inversion strength weakness, more specific and targeted rehabilitation needs to be done.

To our knowledge, this is the first comprehensive study to investigate the long-term effectiveness and its related factors of sprain occurrence for CAI patients with isolated grade III ligament injury. The strength of this study included a relatively long-term follow-up, a detailed systematic balance training protocol and a comprehensive evaluation of the patients and the objective assessment on the postural control improvement in terms of plantar pressure and TTB. It was important to note that although some improvements seem not to last over time, the rehabilitation for the severe type of CAI patients were still effective in the long term. Our results provided more evidence for the use of balance training in CAI cases and would help to optimize rehabilitation plans for this condition. Those with high-risk factors might be prompted to receive early and comprehensive treatment, including the consideration of surgery.

There were still some limitations of the present study. Firstly, the sample size was relatively small in the sprain recurrence group although overall participants enrolled in this study was calculated by the sample size, larger sample are needed to be included in the future research. Secondly, this study focused on patients with lateral ligament injury only while other ligament and syndesmosis injuries also impeded rehabilitation outcomes. So, our results were only suitable for the isolated lateral ankle ligament injury, the balance training for more complex injury type are needed in the future. Thirdly, the patients selected a self-chosen, comfortable walking speed to accomplish the walking task, which might affect the plantar pressure results.

## Conclusion

For the grade III CAI patients with isolated lateral ankle ligament injury, the 3 months’ supervised balance training program significantly improved FAAM scores, foot pressure distribution, static postural control, and muscle strength. However, the effectiveness may be partial declined after 6 months and 1 year. Additional strength exercises for dorsiflexion and eversion should be supplemented from 6 months. The high Beighton score and the initial inversion muscle strength weakness might be the potential risk factors of sprain recurrence after balance training.

## Supplementary Information

**Additional file 1: Appendix 1**. Univariate analysis between sprain recurrence individuals (SR) and control individuals (C). PF, plantarflexion; DF, dorsiflexion; EV, eversion; IV, inversion; HM, Medial heel; HL, lateral heel, M1 to M5, the 1st to 5th metatarsal heads and T1, the hallux.

## Data Availability

The datasets used and/or analysed during the current study are available from the corresponding author on reasonable request.

## Abbreviation

CAI: Chronic ankle instability
ATFL: Anterior talofibular ligament
CFL: Calcaneofibular ligament
OCLs: Osteochondral lesions
COP: Center-of pressure
TTB: Time-to-boundary
gJHM: Generalized joint hypermobility
HM: Medial heel
HL: Lateral heel
M1 ~ M5: 1st to 5th metatarsal heads
T1: Toes
TPF: Time to peak force
FAAM: Foot and ankle ability measure
CAIT: Cumberland ankle Instability tool
TTBML (AP)m: Mean value of the minimum time to boundary in the medial-lateral (anterior-posterior) direction
TTBML (AP)a: Absolute minimum time to boundary in the medial-lateral (anterior-posterior) direction
PF: Plantarflexion
DF: Dorsiflexion
EV: Eversion
IV: Inversion
SR: Sprain recurrence individuals
C: Control individuals

## References

[1] Hertel J (2002). Functional Anatomy, Pathomechanics, and Pathophysiology of Lateral Ankle Instability. J Athl Train.

[2] Hertel J, Corbett RO (2019). An Updated Model of Chronic Ankle Instability. J Athl Train.

[3] Lynch SA, Renstrom PA (1999). Treatment of acute lateral ankle ligament rupture in the athlete. Conservative versus surgical treatment. Sports Med.

[4] Jiang D, Ao YF, Jiao C (2018). Concurrent arthroscopic osteochondral lesion treatment and lateral ankle ligament repair has no substantial effect on the outcome of chronic lateral ankle instability. Knee Surg Sports Traumatol Arthrosc.

[5] Hua Y, Chen S, Li Y (2010). Combination of modified Brostrom procedure with ankle arthroscopy for chronic ankle instability accompanied by intra-articular symptoms. Arthroscopy.

[6] Ferkel RD, Chams RN (2007). Chronic lateral instability: arthroscopic findings and long-term results. Foot Ankle Int..

[7] Kosik KB, McCann RS, Terada M (2017). Therapeutic interventions for improving self-reported function in patients with chronic ankle instability: a systematic review. Br J Sports Med.

[8] Wright CJ, Linens SW (2017). Patient-reported efficacy 6 months after a 4-week rehabilitation intervention in individuals with chronic ankle instability. J Sport Rehabil.

[9] Kim T, Kim E, Choi H (2017). Effects of a 6-week neuromuscular rehabilitation program on ankle-Evertor strength and postural stability in elite women field hockey players with chronic ankle instability. J Sport Rehabil.

[10] Hertel J, Olmsted-Kramer LC (2007). Deficits in time-to-boundary measures of postural control with chronic ankle instability. Gait Posture.

[11] Becker H, Rosenbaum D, Claes L (1997). Measurement of plantar pressure distribution during gait for diagnosis of functional lateral ankle instability. Clin Biomech (Bristol, Avon).

[12] Jackson BC, Medina RT, Clines SH, et al. The Effect of Fibular Reposition Taping on Postural Control in Individuals With Chronic Ankle Instability: A Critically Appraised Topic. J Sport Rehabil. 2018:1–6. 10.1123/jsr.2017-0166. 10.1123/jsr.2017-0166 28952859

[13] Gribble PA, Delahunt E, Bleakley CM (2014). Selection criteria for patients with chronic ankle instability in controlled research: a position statement of the International Ankle Consortium. J Athl Train.

[14] Vuurberg G, Hoorntje A, Wink LM (2018). Diagnosis, treatment and prevention of ankle sprains: update of an evidence-based clinical guideline. Br J Sports Med.

[15] Konradsen L, Holmer P, Sondergaard L (1991). Early mobilizing treatment for grade III ankle ligament injuries. Foot Ankle.

[16] Walker JJ (1989). Surgical treatment of chronic lateral instability of the ankle joint--a new procedure. Am J Sports Med.

[17] Cox JS. Surgical and nonsurgical treatment of acute ankle sprains. Clin Orthop Relat Res. 1985:118–26. 4028542

[18] Hertel J, Olmsted-Kramer LC, Challis JH (2006). Time-to-boundary measures of postural control during single leg quiet standing. J Appl Biomech.

[19] Huang PY, Lin CF, Kuo LC, Liao JC (2011). Foot pressure and center of pressure in athletes with ankle instability during lateral shuffling and running gait. Scand J Med Sci Spor.

[20] Nyska M (2003). Dynamic force distribution during level walking under the feet of patients with chronic ankle instability. Brit J Sport Med.

[21] Kaminski TW, Buckley BD, Powers ME (2003). Effect of strength and proprioception training on eversion to inversion strength ratios in subjects with unilateral functional ankle instability. Br J Sports Med.

[22] Hale SA, Fergus A, Axmacher R (2014). Bilateral improvements in lower extremity function after unilateral balance training in individuals with chronic ankle instability. J Athl Train.

[23] Hale SA, Hertel J, Olmsted-Kramer LC (2007). The effect of a 4-week comprehensive rehabilitation program on postural control and lower extremity function in individuals with chronic ankle instability. J Orthop sports Phys Ther.

[24] Powden CJ, Hoch JM, Jamali BE, Hoch MC. A 4-Week Multimodal Intervention for Individuals With Chronic Ankle Instability: Examination of Disease-Oriented and Patient-Oriented Outcomes. J Athl Train. 2018;54, 384:–396. 10.4085/1062-6050-344-17. 10.4085/1062-6050-344-17 PMC6522089 30589387

[25] Powden CJ, Hoch JM, Hoch MC (2017). Rehabilitation and improvement of health-related quality-of-life detriments in individuals with chronic ankle instability: a Meta-analysis. J Athl Train.

[26] Smith BI, Curtis D, Docherty CL (2018). Effects of hip strengthening on neuromuscular control, hip strength, and self-reported Functional deficits in individuals with chronic ankle instability. J Sport Rehabil.

[27] Hall EA, Docherty CL, Simon J (2015). Strength-training protocols to improve deficits in participants with chronic ankle instability: a randomized controlled trial. J Athl Train.

[28] Linens SW, Ross SE, Arnold BL, Gayle R, Pidcoe P (2014). Postural-stability tests that identify individuals with chronic ankle instability. J Athl Training.

[29] Halabchi F, Angoorani H, Mirshahi M (2016). The Prevalence of Selected Intrinsic Risk Factors for Ankle Sprain Among Elite Football and Basketball Players. Asian J Sports Med.

[30] Witchalls J, Blanch P, Waddington G (2012). Intrinsic functional deficits associated with increased risk of ankle injuries: a systematic review with meta-analysis. Br J Sports Med.

[31] Petrera M, Dwyer T, Theodoropoulos JS (2014). Short- to Medium-term Outcomes After a Modified Brostrom Repair for Lateral Ankle Instability With Immediate Postoperative Weightbearing. Am J Sports Med.

[32] Ryan L (1994). Mechanical stability, muscle strength and proprioception in the functionally unstable ankle. Aust J Physiother.

[33] Munn J, Beard DJ, Refshauge KM (2003). Eccentric muscle strength in functional ankle instability. Med Sci Sports Exerc.

[34] Wilkerson GB, Pinerola JJ, Caturano RW (1997). Invertor vs. evertor peak torque and power deficiencies associated with lateral ankle ligament injury. J Orthop Sports Phys Ther.

[35] Holmes A, Delahunt E (2009). Treatment of common deficits associated with chronic ankle instability. Sports Med.

