# Supplementary material for: The effectiveness and sustainability of supervised balance training in chronic ankle instability with grade III ligament injury: a one-year prospective study
Source: J Foot Ankle Res. 2022 Feb 1;15:9. doi: 10.1186/s13047-022-00514-x (PMC8805278; doi:10.1186/s13047-022-00514-x)
Supplement: Supplementary file 1 — Additional file 1: Appendix 1. Univariate analysis between sprain recurrence individuals (SR) and control individuals (C). PF, plantarflexion; DF, dorsiflexion; EV, eversion; IV, inversion; HM, Medial heel; HL, lateral heel, M1 to M5, the 1st to 5th metatarsal heads and T1, the hallux. [file 13047_2022_514_MOESM1_ESM.docx]

| Variables | SR (N=4) | C(N=16) | T | P |
| --- | --- | --- | --- | --- |
| Muscle strength, N*m/kg |  |  |  |  |
| 120°/s PF | 21.30±5.47 | 21.87±4.14 | 0.76 | 0.865 |
| 120°/s DF | 10.63±2.12 | 8.99±1.84 | 1.06 | 0.255 |
| 120°/s EV | 18.00±1.92 | 18.61±1.39 | 0.19 | 0.587 |
| 120°/s IV | 17.03±4.32 | 17.73±2.09 | 0.17 | 0.731 |
| 60°/s PF | 30.70±0.26 | 32.53±5.60 | 0.84 | 0.601 |
| 60°/s DF | 11.67±3.85 | 8.89±3.21 | 0.28 | 0.272 |
| 60°/s EV | 18.17±3.95 | 17.00±3.02 | 2.08 | 0.621 |
| 60°/s IV | 13.47±1.10 | 18.27±2.09 | -6.44 | 0.014* |
| Demographic data |  |  |  |  |
| Beighton | 6.33±1.53 | 1.43±1.62 | 3.33 | 0.012* |
| Injury time, month | 7.67±1.15 | 7.89±3.14 | 0.41 | 0.251 |
| Height, cm | 161.0±4.58 | 169.57±7.8 | -1.73 | 0.124 |
| Age, year | 30.0±12.17 | 26.57±10.2 | 0.66 | 0.661 |
| Weight, kg | 57.3±4.9 | 62.00±10.3 | -0.72 | 0.497 |
| BMI, kg/m^2^ | 21.7±6.3 | 21.5±3.4 | 0.25 | 0.968 |
| Standardized Peak force (N/kg) |  |  |  |  |
| M1 | 0.77±0.54 | 2.55±1.05 | -1.31 | 0.035* |
| M2 | 4.08±1.84 | 4.14±1.30 | -1.05 | 0.954 |
| M3 | 3.90±1.27 | 2.79±1.23 | 0.9 | 0.233 |
| M4 | 2.00±0.93 | 1.05±0.50 | 1.23 | 0.202 |
| M5 | 1.29±0.42 | 0.86±0.78 | 0.32 | 0.415 |
| HL | 3.53±1.18 | 5.47±1.21 | 4.66 | 0.045* |
| HM | 2.44±0.29 | 4.31±1.34 | 4.52 | 0.041* |
| T1 | 0.75±0.12 | 1.77±1.26 | -1.16 | 0.217 |
| Time to peak force (%) |  |  |  |  |
| M1 | 66.1±14.2 | 66.6±6.9 | -1.35 | 0.952 |
| M2 | 72.1±5.8 | 74.9±5.7 | -0.6 | 0.486 |
| M3 | 69.5±4.1 | 70.9±6.4 | -0.67 | 0.732 |
| M4 | 62.5±13.4 | 58.5±13.9 | 0.59 | 0.685 |
| M5 | 50.1±12.7 | 43.6±10.1 | 1.09 | 0.429 |
| HL | 12.9±8.0 | 14.4±5.4 | -0.77 | 0.721 |
| HM | 15.8±1.6 | 13.2±6.8 | 0.35 | 0.5462 |
| T1 | 71.8±4.1 | 75.9±12.1 | -0.70 | 0.598 |
| Time to boundary |  |  |  |  |
| Min Medial-Lateral | 0.09±0.01 | 0.08± 0.02 | 1.01 | 0.328 |
| Min Anterior-Posterior | 0.63±0.25 | 0.67±0.45 | 0.11 | 0.907 |
| Mean Medial-Lateral | 1.77±0.25 | 1.62±0.40 | -1.14 | 0.575 |
| Mean Anterior-Posterior | 3.17 ±0.65 | 3.15±0.56 | 0.64 | 0.966 |
| SD Medial-Lateral | 1.75 ±0.27 | 1.73± 0.13 | 1.86 | 0.846 |
| SD Anterior-Posterior | 2.16 ±0.24 | 2.18 ±0.30 | -0.01 | 0.914 |
| COP velocity(cm/s) |  |  |  |  |
| Medial-Lateral | 4.59 ±1.30 | 5.37 ±1.74 | 0.93 | 0.512 |
| Anterior-Posterior | 6.26± 2.41 | 6.55 ±1.71 | 0.76 | 0.832 |
